# Sex and age differences in cognitive presentation, mild cognitive impairment, and dementia prevalence in older patients with concussion – a retrospective observational study

**DOI:** 10.1002/alz70857_106729

**Published:** 2025-12-26

**Authors:** Davina Kavita Premraj, Yasmin Soliman, Carmela Tartaglia, Charles Tator, Artee Srivastava

**Affiliations:** ^1^ Krembil Discovery Tower, Tanz Centre for Neurodegenerative Disease, Concussion and Memory Clinic, Toronto, ON, Canada; ^2^ UHN, Toronto, ON, Canada; ^3^ Canadian Concussion Centre, Krembil Brain Institute, University Health Network, Toronto, ON, Canada; ^4^ Canadian Concussion Centre, Toronto Western Hospital, Krembil Brain Institute, University Health Network, Toronto, ON, Canada

## Abstract

**Background:**

Falls and concussions are a growing concern among aging populations. Often, older adults with concussions are not properly screened for post‐concussion symptoms and can be diagnosed with mild cognitive impairment (MCI) or dementia. Additionally, there may be sex differences related to cognitive presentation. Ultimately, recognizing sex differences in cognitive presentation following concussion is essential for improving concussion management in older adults.

**Method:**

Patients aged >50 years with concussion who were seen at the Concussion Clinic at the Toronto Western Hospital with a concussion in the last three years were included in this study. All data were systematically collected using REDCap (Research Electronic Data Capture). All participating patients provided informed consent for their data to be used for research purposes. The dataset includes demographic details, clinical diagnoses, and cognitive assessments, including the Toronto Cognitive Assessment (TorCA) across adults with concussions within the past three years. After grouping by sex, patients were further subdivided into three categories: Normal, Mild Cognitive Impairment (MCI), and Dementia based on TorCA score, MCI probability, and age.

**Result:**

Significantly more females (*n* = 50; mean age = 63.7) than males (*n* = 26; mean age = 60.5) presented with a concussion diagnosis within three years of the appointment date than males (*n* = 24) within the last three years (*p* <0.05). When comparing diagnoses, although not significant (*p* = 0.12) fewer females with concussion had mild cognitive impairment (6%) compared with males (21%) while dementia frequency was similar (females 35%; males 38%). Dementia prevalence was higher in adults aged 65+ (48.15%) compared with those aged 50‐64 (28.26%), though not statistically significant (*p* = 0.144). The TORCA scores were not significantly different between males (256.5) and females (274.2), *p*‐val=0.11.

**Conclusion:**

In older adults, there is a higher prevalence of persisting symptoms of concussion in females compared to males, similar to what is known in younger populations. As expected, dementia was more frequent in the older group. Further investigation is required to elucidate the role of concussion in patients who are diagnosed with MCI or dementia when presenting with persisting symptoms of concussion.

